# Dynamic contrast-enhanced breast MRI at 7T and 3T: an intra-individual comparison study

**DOI:** 10.1186/s40064-015-1654-7

**Published:** 2016-01-05

**Authors:** Gisela L. G. Menezes, Bertine L. Stehouwer, Dennis W. J. Klomp, Tijl A. van der Velden, Maurice A. A. J. van den Bosch, Floortje M. Knuttel, Vincent O. Boer, Wybe J. M. van der Kemp, Peter R. Luijten, Wouter B. Veldhuis

**Affiliations:** Department of Radiology and Nuclear Medicine, University Medical Centre Utrecht, P.O. Box 85500, 3508 GA Utrecht, The Netherlands

**Keywords:** 7T, 3T, Breast MRI, Breast cancer, BI-RADS, Ultra-high field MRI

## Abstract

The aim of this study is to compare the current state of lesion identification, the BI-RADS classification and the contrast-enhancement behavior at 7T and 3T breast MRI in the same patient group. Twenty-seven patients with thirty suspicious lesions were selected for this prospective study and underwent both 7T and 3T MRI. All examinations were rated by two radiologists (R1 and R2) independently on image quality, lesion identification and BI-RADS classification. We assessed sensitivity, specificity, NPV and PPV, observer agreement, lesion sizes, and contrast-enhancement-to-noise ratios (CENRs) of mass lesions. Fifteen of seventeen histopathological proven malignant lesions were detected at both field strengths. Image quality of the dynamic series was good at 7T, and excellent at 3T (P = 0.001 for R1 and P = 0.88 for R2). R1 found higher rates of specificity, NPV and PPV at 7T when compared to 3T, while R2 found the same results for sensitivity, specificity, NPV and PPV for both field strengths. The observers showed excellent agreement for BI-RADS categories at 7T (κ = 0.86) and 3T (κ = 0.93). CENRs were higher at 7T (P = 0.015). Lesion sizes were bigger at 7T according to R2 (P = 0.039). Our comparison study shows that 7T MRI allows BI-RADS conform analysis. Technical improvements, such as acquisition of T2w sequences and adjustment of B1+ field inhomogeneity, are still necessary to allow clinical use of 7T breast MRI.

## Background

Dynamic contrast-enhanced magnetic resonance imaging (DCE-MRI) of the breast has become a well-established imaging method for detection of breast carcinomas, and an increased use of 1.5 tesla (T) and 3T DCE-MRI systems has been observed over the past decades. Compared to conventional mammography and ultrasound, DCE-MRI is more accurate in detecting multifocal, multicentric and contralateral disease, in assessing the response to neoadjuvant chemotherapy and in providing preoperative staging (Peters et al. 2008). When compared to 1.5T, a higher field strength (3T) showed to have increased signal-to-noise-ratio (SNR), higher spatial and temporal resolution (Rahbar et al. 2013, 2015). The 3T field also showed differential effects on T1 relaxation times of non-enhancing compared to gadolinium-enhancing tissue, which results in better contrast resolution of the enhancing lesions (Rahbar et al. 2013, 2015; Soher 2007). Recently, there has been a growing interest in investigating the potential role of 7T MRI in breast cancer diagnosis and management (Menezes et al. 2014; Pinker et al. 2014; Stehouwer et al. 2013b; Umutlu et al. 2011). Moving to 7T not only increases SNR (Brown et al. 2013; Korteweg et al. 2011) (and also spatial resolution), (van de Bank et al. 2013) but also brings a greater spectral dispersion, significantly improving magnetic resonance spectroscopy (MRS) (Klomp et al. 2011). Considering these new possibilities, the focus of breast imaging research at 7T is not only improving morphology assessment of breast lesions, but also moving towards obtaining metabolic and cellular information (Klomp et al. 2011; Loo et al. 2011; van der Kemp 2014)

There are also drawbacks of 7T, such as a greater heterogeneity of the static magnetic field (B0) and of the applied RF field (B1+). Furthermore, breast coils had to be developed since none were commercially available a decade ago when 7T became available for whole-body imaging. In literature the first attempt to perform DCE-MRI breast imaging at 7T was made by Umutu et al. using a single loop coil, showing the complexity of ultra–high field breast MRI. Only moderate image quality was achieved when using this coil (Umutlu et al. 2011). Subsequent improvements in hardware and imaging strategies have led to an improved image quality and showed that 7T breast MRI is amenable to BI-RADS lexicon conform analysis (Stehouwer et al. 2013a, b). Nowadays, the first bilateral set-ups are available, which illustrates the evolution of 7T towards clinical usage (Brown et al. 2014; Gruber et al. 2014; Pinker et al. 2014; Stehouwer et al. 2013a; van der Velden 2014). Apart from the new contrast mechanisms available at 7T, such as chemical exchange saturation transfer (CEST) and ^31^phosphorous magnetic resonance spectroscopy (^31^P-MRS), the conventional breast MRI still needs to at least offer comparable imaging results to the current clinical standard of 3T imaging to maintain good medical care.

Comparisons of 7T versus breast imaging at lower field strengths are scarce. So far, studies with healthy volunteers imaged using T1-weighted (T1w) non contrast-enhanced imaging presented similar or better results for 7T. The 7T images showed increased SNR, better fat–water contrast measures and better objective image quality scores (Brown et al. 2013; Umutlu et al. 2011; van der Velden 2014). In a patient setting, using DCE-MRI, comparisons between 7T and 3T fields have been made only in one patient case (Stehouwer et al. 2013a), and in one patient study (Brown et al. 2013). These papers suggest that DCE-MRI at 7T is technically feasible (Stehouwer et al. 2013a), and it can provide a high sensitivity and specificity using high temporal and spatial resolution imaging (Brown et al. 2013).

The aim of this study is to compare the current state of lesion identification and BI-RADS classification at DCE-MRI at 7T and 3T in the same patient group.

## Results

### Study population

Thirty breast lesions were reported in 27 patients. All lesions were detected on conventional imaging, and were classified as BI-RADS 4 (n = 21) and BI-RADS 5 (n = 9). The mean age was 55 years (SD 8; range 32–74 years). Of the 30 index lesions, 17 were histopathologically malignant, and 13 were benign (Table 1).

**Table 1:** Characteristics of the 27 patients with 30 suspicious breast lesions detected on conventional breast imaging

Characteristics	
Age (years)	Mean: 55 (SD: 8, range: 32–74)
Presentation on conventional imaging	
Mass	15 (50 %)
Calcifications	11 (37 %)
Mass + calcifications	1 (3 %)
Architectural distortion	1 (3 %)
Architectural distortion + calcifications	2 (7 %)
Lesion size on conventional imaging (mm)	mean: 19 (SD: 9, range: 7–39)^a^
BI-RADS category on conventional imaging per patient	
4	21 (70 %)
5	9 (30 %)
Histological type	
Malignant	17 (57 %)
Invasive ductal carcinoma	5
Invasive lobular carcinoma	1
Invasive ductulolobular carcinoma	7
Ductal carcinoma in situ with invasive component	2
Ductal carcinoma in situ	2
Non-malignant	13 (43 %)
Columnar cell lesion	2
Radial scar lesion	1
Atypical ductal hyperplasia	1
Cyst	1
Hamartoma	1
Fibroadenoma	2
Apocrine metaplasia	2
Fibrocystic changes	2
Sclerosing adenosis	1

^a^Lesion size of the 15 mass lesions

In 8 patients the 7T scan was conducted using a unilateral breast coil and in 19 using a bilateral breast coil. Fifteen women were postmenopausal. The remaining 11 patients were imaged with a mean of 3.4 days between examinations.

### Image assessment

**Fig. 1:**
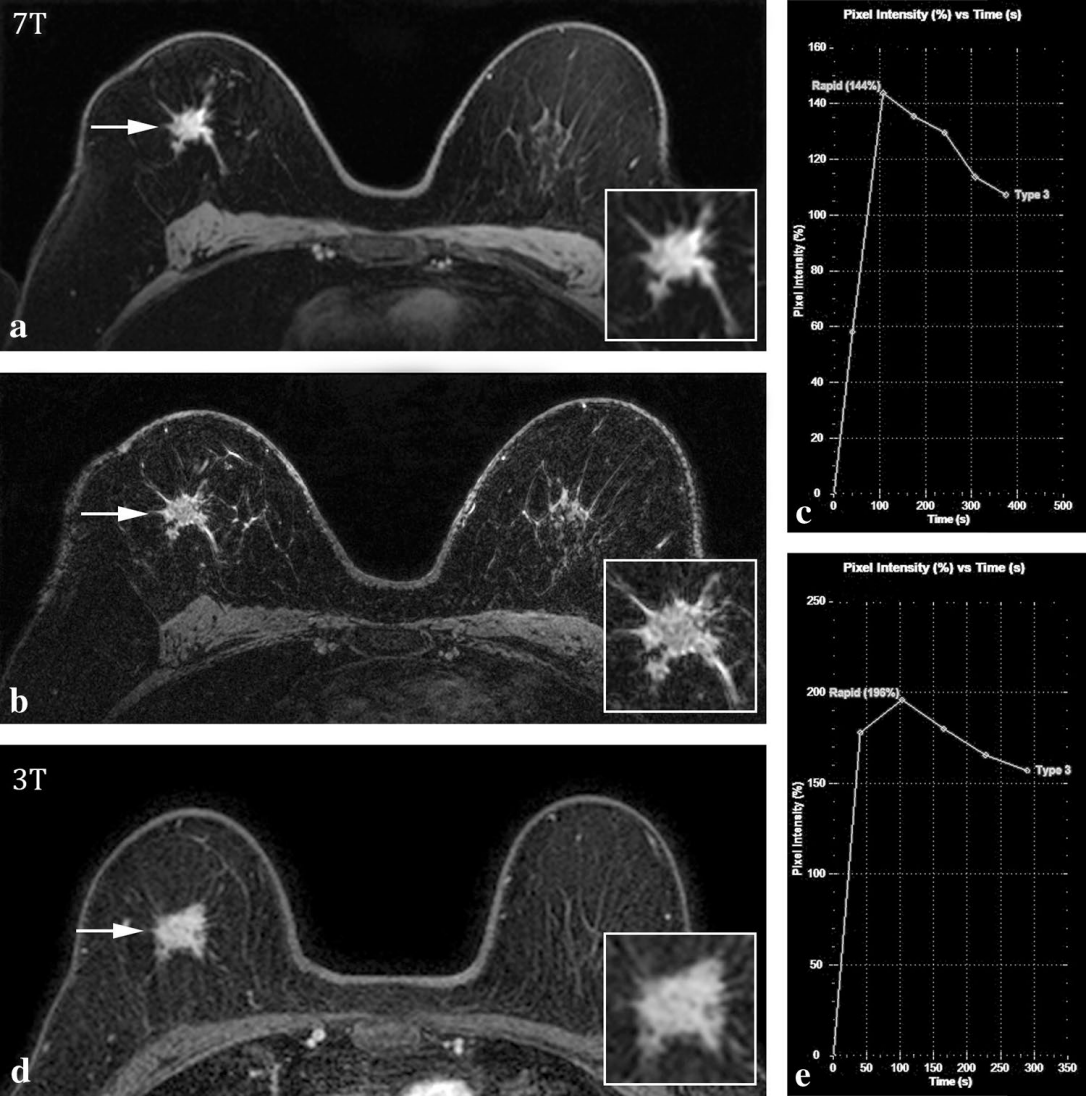
7T (**a**–**c**) and 3T (**d**, **e**) MRI images of a 67-year-old female with an invasive lobular carcinoma in her right breast. Transverse image of 2nd post contrast-injection series (**a**, **d**) shows an irregular mass lesion with spiculated margins (*arrows*) on both field strengths. *Inset* shows zoomed-in image. Ultra-high resolution 7T image of the same slice (**b**). The kinetic curve assessment showed an initial rapid rise and wash-out pattern in the delayed phase on both field strengths (**c**, **e**). Both observers rated the lesion as BI-RADS 5

Both observers detected, at both field strengths, 15 of 17 malignant index. Both observers classified 11 lesions as mass, 2 as non-mass-like-enhancement and 2 as architectural distortions without enhancement. One pure DCIS and one IDC were not detected at either field strength. The detected malignant index lesions and BI-RADS descriptors are presented in Table 2. The table also illustrates the similarities and differences in ratings between field-strengths for both observers. Figure 1 shows one example of a patient with a malignant lesion.

**Table 2:** Histopathological proven malignant index lesions descriptors at both field strengths

Case	Field	Shape	Margin	Enhancement	Initial rise	Delayed phase	Category
1	7T	Irregular	Spiculated	Heterogeneous/homogeneous	Rapid	Wash-out	5
	3T^a^	Irregular	Spiculated	Rim/heterogeneous	Rapid	Wash-out	5
2	7T	Irregular	Spiculated	Heterogeneous	Rapid	Wash-out	5
	3T^a^	Irregular	Spiculated	Heterogeneous/homogeneous	Rapid/medium	Plateau	5/4
3	7T	Irregular	Spiculated/irregular	Heterogeneous	Rapid	Wash-out	5
	3T^a^	Irregular	Irregular	Heterogeneous	Rapid	Wash-out	5
4	7T	Irregular	Spiculated	Heterogeneous	Rapid	Wash-out	5
	3T^a^	Irregular	Spiculated	Rim enhancement	Rapid	Wash-out	5
5	7T	Irregular	Spiculated/irregular	Heterogeneous	Rapid	wash-out	5
	3T^a^	Irregular/oval	Spiculated	Heterogeneous/rim	Rapid	Wash-out	5
6	7T	Irregular	Spiculated	Heterogeneous	Rapid	Wash-out	5
	3T	Irregular	Spiculated	Heterogeneous	Rapid	Wash-out	5
7	7T	Irregular	Spiculated	Heterogeneous	Rapid	Wash-out	5
	3T	Irregular	Spiculated	Heterogeneous	Rapid	Wash-out	5
9	7T	Irregular	Spiculated	Heterogeneous	Rapid	Wash-out	5
	3T	Irregular	Spiculated	Heterogeneous	rapid	Wash-out	5
18	7T	Irregular	Spiculated	Heterogeneous	Rapid	Wash-out	5
	3T	Irregular	Spiculated	Heterogeneous	Rapid	Wash-out	5
23	7T	Irregular	Spiculated	Heterogeneous	Rapid	Wash-out	5
	3T	Irregular	Spiculated	Heterogeneous	Rapid	Wash-out	5
25	7T	Irregular	Spiculated	Heterogeneous	Rapid	Wash-out	5
	3T	Irregular	Spiculated	Heterogeneous	Rapid	Wash-out	5

		Non-mass-like enhancement distribution		Internal enhancement			Category
14	7T	Segmental/ductal		Clumped			4/5
	3T	segmental/ductal		Clumped			4/5
22	7T	Segmental		Heterogeneous			4
	3T	Segmental		Heterogeneous			4
		Architectural distortion					
26^b^	7T	Architectural distortion		No enhancement			4
	3T	Architectural distortion		No enhancement			4

^a^Ratings by R1 and R2. In case of a discrepancy between observers, both ratings are displayed separated by a slash. In case of a discrepancy between field strengths the case is marked with superscript a

^b^Two malignant lesions in the same patient were described as architectural distortions

All patients and identified lesions are presented in Table 3 (with their respective BI-RADS classification and histopathological results).

**Table 3:** Identified mass and non-mass-like enhancement lesions at 7T and 3T plus BI-RADS classification and pathology results

Case	R1-7T	BI-RADS	R2-7T	BI-RADS	R1-3T	BI-RADS	R2-3T	BI-RADS	Pathology
1	Mass	5	Mass	5	Mass	5	Mass	5	IDLC
2	Mass	5	Mass	5	Mass	5	Mass	4	IDLC
3	Mass	5	Mass + non-mass	5	Mass	5	Mass + non-mass	5	IDLC
4	Mass	5	Mass	5	Mass	5	Mass	5	IDLC
5	Mass + satellite	5	Mass	5	Mass	5	Mass	5	IDC
6	Mass	5	Mass	5	Mass	5	Mass	5	IDC
7	Mass	5	Mass	5	Mass	5	Mass	5	IDLC
8	Mass	4	Mass	4	Mass	4	Mass	5	Radial scar lesion
9	Mass + satellite	5	Mass + non-mass	5	Mass + non-mass	5	Mass	5	IDLC
10	No lesion	1	No lesion	2	No lesion	1	No lesion	2	Apocrine metaplasia
11	No lesion	3	*Non*-*mass*	*2*	*Non*-*mass*	*3*	*Non*-*mass*	*3*	Cyst
12	*Mass*	*3*	Non-mass	4	Non-mass	4	Mass	5	Fibrocystic changes
13	No lesion	2	No lesion	2	No lesion	1	No lesion	2	DCIS
14	Non-mass	4	Non-mass	5	Non-mass	4	Non-mass	5	DCIS + invasive component
15	No lesion	1	No lesion	*2*	*Non*-*mass*	*3*	No lesion	2	Hamartoma
16	*Mass*	*2*	*Mass 2*×	*3*	No lesion	2	*Mass 2*×	*3*	Apocrine metaplasia
17	Mass 2× + *non*-*mass*	*3*	Mass 2×	2	2× Mass	3	Mass 2×	3	Fibrocystic changes + fibroadenoma
18	Mass + non-mass	5	Mass	5	2× Mass	5	Mass	5	ILC
19	Non-mass	4	Non-mass	3	Mass + non-mass	4	Non-mass 2×	3	Columnar cell lesion
20	No lesion	1	No lesion	2	No lesion	2	No lesion	2	Columnar cell lesion + ADH
21	No lesion	2	no lesion	2	No lesion	2	No lesion	2	Sclerosing adenosis
22	Non-mass	4	Non-mass	4	Non-mass	4	Non-mass	4	DCIS + invasive component
23	Mass	5	Mass	5	Mass	5	Mass	5	DCIS
24	No lesion	2	No lesion	2	No lesion	2	No lesion	2	IDC
25	Mass	5	Mass	5	Mass	5	Mass	5	IDC
26	Architectural distortion 2×	4	Architectural distortion 2×	4	Architectural distortion 2×	4	Architectural distortion 2×	4	IDC + IDLC
27	No lesion	2	No lesion	2	No lesion	2	No lesion	2	Fibroadenoma

Additional findings are marked in italic

**Fig. 2:**
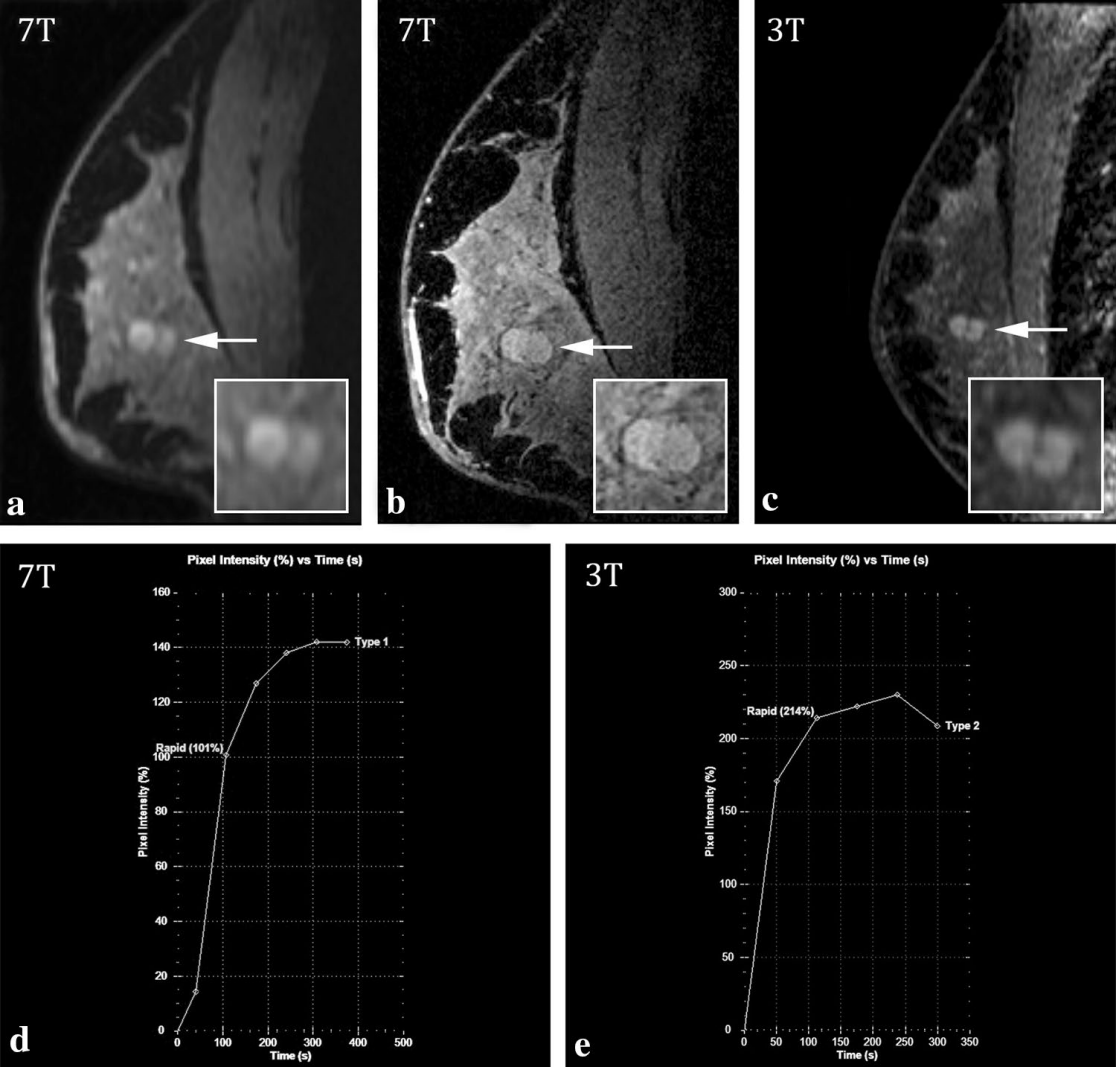
7T (**a**, **b**, **d**) and 3T (**c**, **e**) MRI images of a 65-year-old female patient. The depicted lesion in her right breast was diagnosed as fibrocystic changes after biopsy. Sagittal images of 2nd post contrast-injection series (**a**, **c**) show a lobular lesion (*arrow*) with irregular (R1 at 3T) or smooth margins (R1 at 7T and R2 at 3T and 7T). *Inset* shows zoomed-in image. Ultra-high resolution 7T image of the same slice (**b**). The kinetic curve assessment at 7T shows a rapid rise and persistent pattern in the delayed phase (**d**), and at 3T a rapid rise and plateau pattern (**e**). R1 classified the lesion as BI-RADS 3, and R2 as BI-RADS 3 (3T) and BI-RADS 2 (7T)

In 3 of the 13 non-malignant lesions, a BI-RADS 4 or higher was rated at one or both field strengths. In case number 8 (Table 3), a radial scar lesion was rated as BI-RADS 4 or 5 by both observers at both field strengths. In case number 12, R1 identified a non-mass-like enhancement at 3T (rated as BI-RADS 4). The same lesion was not identified by this observer at 7T. R2 identified a non-mass-like enhancement at 7T (BI-RADS 4), and a mass lesion at 3T (BI-RADS 5). In case number 19, R1 identified a non-mass-like enhancement lesion at 7T (BI-RADS 4), and a mass and non-mass-like enhancement at 3T (also rated as BI-RADS 4). R2 identified non-mass-like enhancement at both field strengths and classified both as BI-RADS 3. The remaining 10 cases with proven benign index lesions had examinations that were correctly classified as benign at both field strengths. Figure 2 shows an example of a benign lesion.

**Fig. 3:**
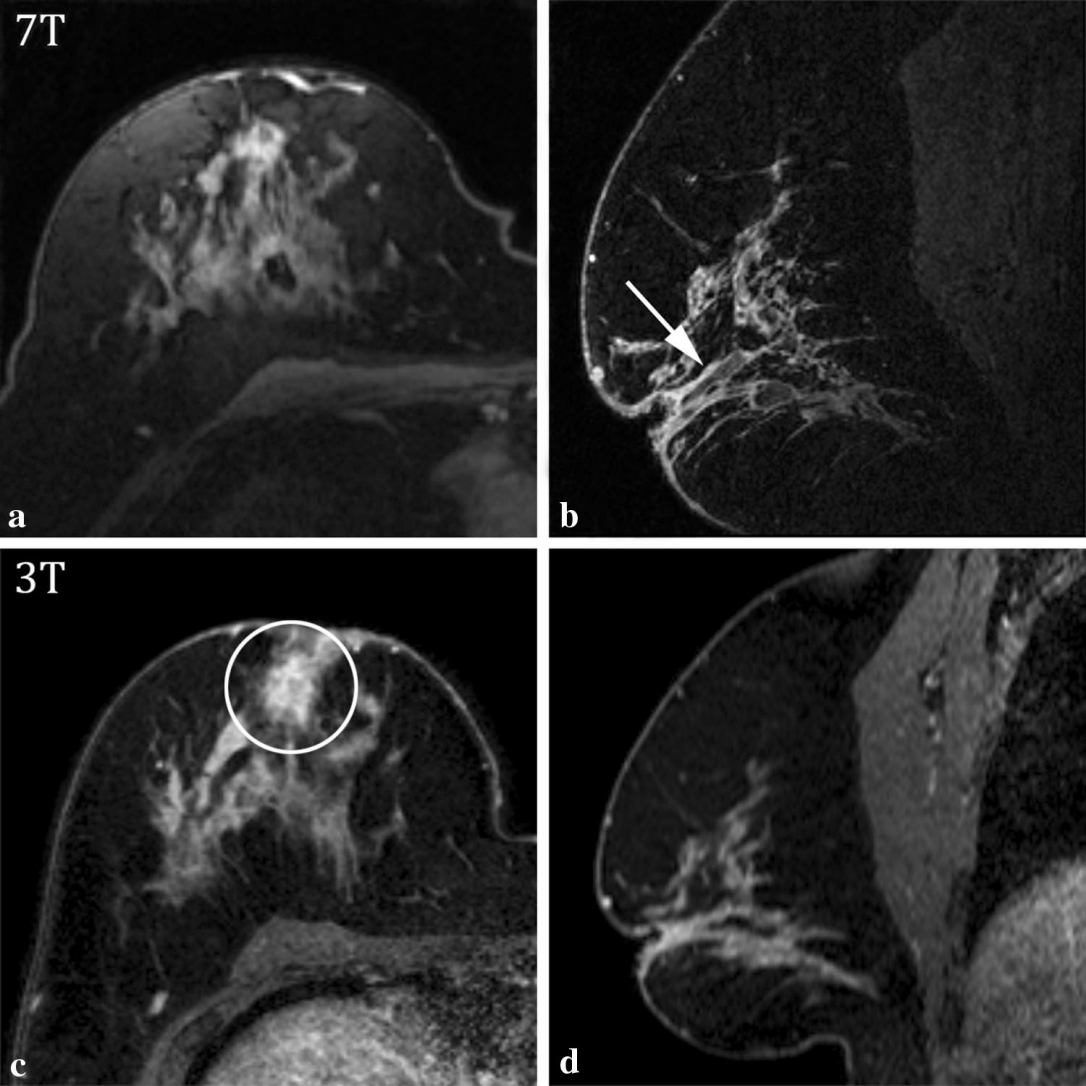
7T (**a**, **b**) and 3T (**c**, **d**) MRI results of a 47-year-old female patient with a history of inverted nipples. The biopsied index lesion in the right breast showed to be a cyst. Transverse image of 2nd post contrast-injection series (**a**, **c**). 7T MRI sagittal slice of high-resolution imaging (**b**), sagittal slice of 3T dynamic series at approximately the same location (**d**). At 7T MRI, diffuse non-mass-like enhancement was identified by R2, while R1 identified periductal enhancement (*arrow*). At 3T MRI, a focal non-mass-like enhancement was identified by R1 (*circle*), and multiple regions of non-mass-like enhancement were seen by R2. The observers rate the images BI-RADS 3 for 3T MRI, and BI-RADS 3 (R1) and 2 (R2) for 7T MRI

Table 3 also shows that a number of additional findings were made independently of the index lesions. These findings (in italic) occurred in five cases, and were all classified as benign. Figure 3 shows an example of benign additional finding (periductal enhancement), classified as BI-RADS 1–3 by both observers. The final histopathological analysis showed a cyst.

According to the first observer (R1), the mean image quality score was 2.14 ± 0.82 for 7T images, and 1.37 ± 0.49 for 3T. The second observer (R2) scored image quality as 1.96 ± 0.65 for 7T, and 1.70 ± 0.60 for 3T (P = 0.001 for R1, and P = 0.88 for R2). Image quality scores were also calculated considering only the scans performed with the bilateral coil. We had similar results: R1 rated the image quality of the dynamic series significantly better at 3T (P = 0.021), and R2 saw no significant difference between fields (P = 0.132). Image quality scores are presented in Table 4.

**Table 4:** Image quality assessment of the 27 dynamic series rated by R1 and R2 at both field strengths

	R1*		R2	
Image quality	7T	3T	7T	3T
Excellent	7	17	6	10
Good	9	10	16	15
Moderate	11	0	5	2
Poor	0	0	0	0
Non-diagnostic	0	0	0	0

* <0.05 Wilcoxon matched pairs signed rank test

Table 5 shows sensitivity, specificity, NPV and PPV at 3T and 7T for both observers. The rates were calculated for the 19 patients with bilateral coil and for the total number of patients (27).

**Table 5:** Sensitivity, specificity, NPV and PPV at 3T and 7T for both observers (R1 and R2)

	Sensitivity	R1 unilateral + bilateral coil (n = 27)^a^		NPV
		Specificity	PPV	
3T	88 % (CI 0.62–0.98)	77 % (CI 0.46–0.94)	83 % (CI 0.58–0.96)	83 % (CI 0.51–0.97)
7T	88 % (CI 0.62–0.98)	85 % (CI 0.54–0.97)	88 % (CI 0.62–0.98)	85 % (CI 0.54–0.97)

	Sensitivity	R1 bilateral coil (n = 19)^b^		NPV
		Specificity	PPV	
3T	80 % (CI 0.44–0.96)	83 % (CI 0.51–0.97)	80 % (CI 0.44–0.96)	83 % (CI 0.51–0.97)
7T	80 % (CI 0.44–0.96)	92 % (CI 0.60–0.99)	89 % (CI 0.51–0.99)	85 % (CI 0.54–0.97)

	Sensitivity	R2 unilateral + bilateral coil (n = 27)^a^		NPV
		Specificity	PPV	
3T	88 % (CI 0.62–0.98)	85 % (CI 0.54–0.97)	88 % (CI 0.62–0.98)	85 % (CI 0.54–0.97)
7T	88 % (CI 0.62–0.98)	85 % (CI 0.54–0.97)	88 % (CI 0.62–0.98)	85 % (CI 0.54–0.97)

	Sensitivity	R2 bilateral coil (n = 19)^b^		NPV
		Specificity	PPV	
3T	80 % (CI 0.44–0.96)	92 % (CI 0.6–0.99)	89 % (CI 0.51–0.99)	85 % (CI 0.54–0.97)
7T	80 % (CI 0.44–0.96)	92 % (CI 0.6–0.99)	89 % (CI 0.51–0.99)	85 % (CI 0.54–0.97)

^a^Scans performed using both coils

^b^Scans performed using only the bilateral coil

Inter-observer agreement for BI-RADS assessment categories was excellent in both 7T (κ = 0.93 and P = 0.0001) and 3T (κ = 0.86 and P = 0.0001).

**Fig. 4:**
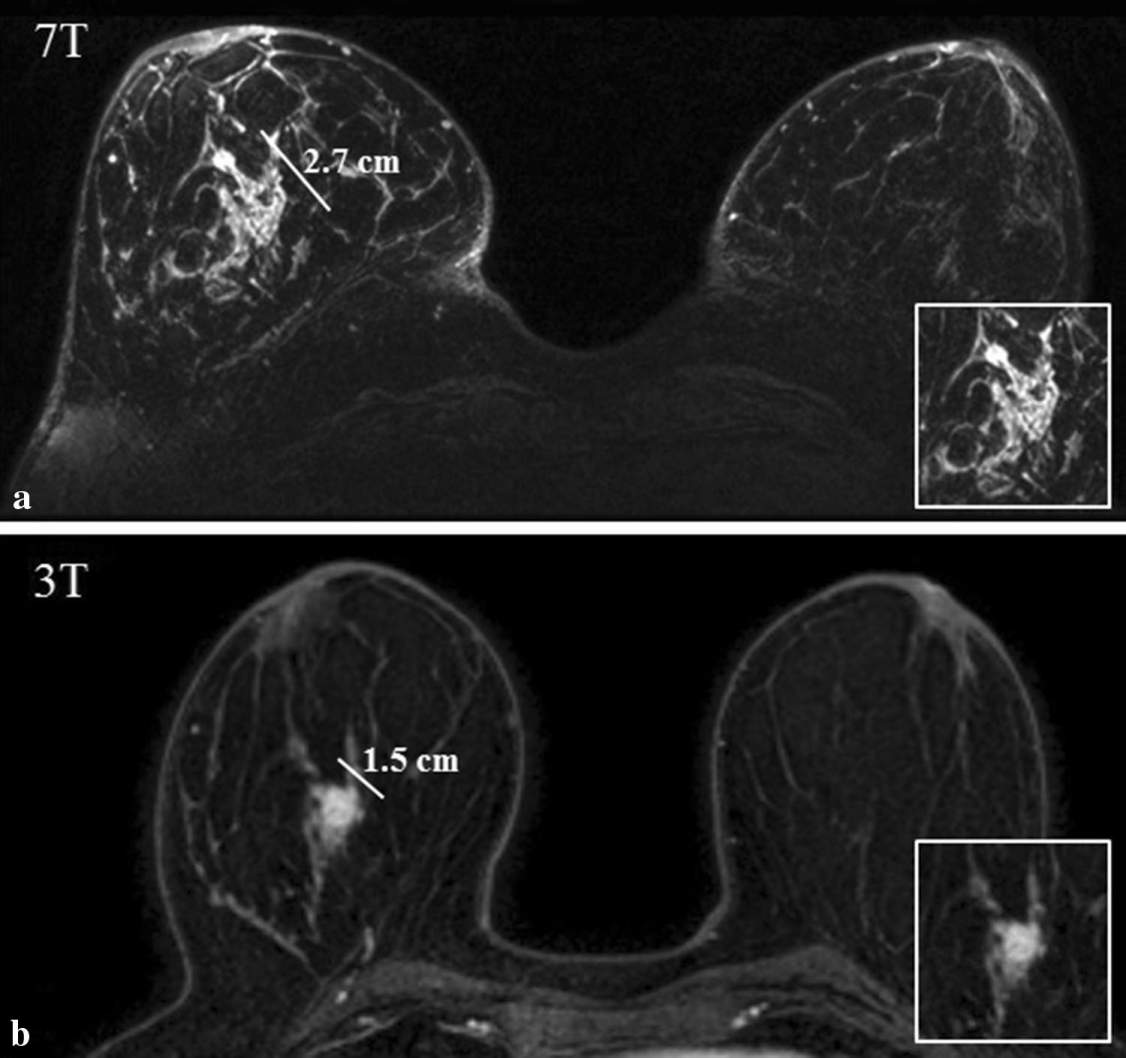
7T (**a**) and 3T (**b**) images of a 52-year-old female patient diagnosed with a ductal carcinoma in her right breast. The morphological characteristics, the size and the borders of the lesion are more clearly depicted in ultra-high resolution 7T image (**a**) when compared to 3T images (**b**). This variation in tumor size has an impact in both staging and treatment of breast tumors. Both observers agreed on the sizes of the lesion

The 11 malignant mass lesions had a mean size of 31 mm at 7T MRI according to R1 (SD 18, range 16–95) and a mean size of 28 mm at 3T MRI (SD 23, range 10–95). For R2 the mean size at 7T was 28 mm (SD 16, range 15–72), and at 3T was 24 mm (SD 13, range 12–58). The differences between field strengths were not statistically significant for R1 (P = 0.864), but according to R2 lesions sizes were significantly bigger at 7T (P = 0.039). Figure 4 shows a patient case in which the size of the tumor was much more clearly depicted at 7T when compared to 3T.

CENRs measurements of the malignant mass lesions showed to be significantly higher at 7T (P = 0.015) with a mean of 2.8 (SD 1.0, range 1.4–4.6), compared to 2.0 at 3T (SD 0.6, range 1.2–3.4).

## Discussion

The performance in this study of both 7T and 3T DCE-MRI (using conventional imaging parameters) is conform literature (Peters et al. 2008). Sensitivity, specificity, NPV and PPV were equal at both field strengths for R2. However, R1 achieved higher specificity, NPV and PPV rates at 7T (considering both coils and only bilateral coil). These results could be related to the simultaneous high spatial and temporal resolution provided at 7T when compared to the resolution obtained at 3T. Our findings may be somewhat limited by the small sample size, though similar results have previously been described. Gruber et al. had similar outcomes to ours. The authors compared bilateral DCE-MRI of the breast at 3T and 7T in 24 patients and the results showed higher sensitivity, PPV and NPV at 7T. They attributed these results to high temporal and spatial resolution provided at the ultra-high magnetic field (Gruber et al. 2014).

In prospective studies, Pinker et al. and Kuhl et al. analyzed the trade-off between temporal and spatial resolution in dynamic post-contrast bilateral MR imaging of the breast. Both authors concluded that, at lower field strengths, the SNR is not sufficient to acquire images at higher spatial resolution, even when applying acceleration techniques to characterize the breast lesions (Kuhl et al. 2005; Pinker et al. 2009). Recently, van den Bank et al. hypothesized that the necessary gain in SNR could be obtained at 7T field, while acceleration could be obtained with high-density receiver coil arrays. Using a unilateral 30-channel receive-only element breast coil (combined with a dual-channel transmit coil) the authors concluded that the high density of receive coil elements allowed high temporal and spatial resolution DCE-MRI of breast at 7T, associated with less DWI distortion (van de Bank et al. 2013).

The ultra-high resolution imaging at 7T performed in our study allowed better morphological characterizations of malignant breast tumors when compared to 3T, as illustrated in Figs. 1 and 4. In Fig. 4 the lesions’ dimensions are more clearly depicted in ultra-high resolution 7T images when compared to 3T. At 3T, the spiculated lesion would be classified as a T1 (tumor is less than 2 cm) and at 7T it would be classified as T2 (between 2 and 5 cm). This difference upgrades the cancer from stage I to stage II, which results in an impact in treatment and, ultimately, in prognosis.

The image quality for the dynamic series at 7T was overall rated good by both observers. Even though R1 achieved higher specificity, PPV and NPV rates at 7T, the same observer rated the image quality of the dynamic series at 3T significantly better, which indicates that there is still room for improvement. Current technical developments, such as dynamic B0 field monitoring and correction (Boer et al. 2012), and RF pulses designed to compensate the linear decreasing B1+ field(van Kalleveen et al. 2015) may improve the image quality at 7T. As an example, Boer et al. developed a simple field probe that proved to be useful to monitor temporal B0 field variations. The acquired temporal B0 field information could drive a dynamic module to correct the B0 magnetic field in real time (Boer et al. 2012). In a recent study, van Kalleveen et al. were able to compensate the inhomogeneous B1+ field (while keeping the specific absorption rate low) by applying tilt optimized flip uniformity RF pulses to the breast surface coil (van Kalleveen et al. 2015).

Brown et al. performed two studies in healthy volunteers, and the results showed equal image quality for non-contrast-enhanced T1w imaging at 7T and 3T MRI (Brown et al. 2013, 2014). In these studies, SPAIR was used as fat suppression technique, which is less prone to B0 field inhomogeneities than a binomial RF pulse. However, SPAIR is incompatible with optimal dynamic imaging at 7T due to specific absorption rate (SAR) limitations. Other fat suppression techniques (such as a DIXON-based method) could be investigated for 7T dynamic imaging (Ma 2008) since it showed promising results at 3T MRI and is less sensitive to B0 distortions (Le-Petross et al. 2010).

In vitro research showed that gadolinium based contrast agents are slightly less effective at 7T compared to 3T (Noebauer-Huhmann et al. 2010). However, our results showed a higher CENRs at 7T, and Gruber et al. also reported a non-significant difference in relative signal-enhancement between 3T and 7T (Gruber et al. 2014). It can thus be suggested that, with adequate T1 weighting and the high SNR available at 7T, the effectiveness of the contrast agent is (more than) sufficient. With the higher CENRs, all invasive mass lesions were conspicuous at 7T.

To our knowledge this is the second cohort study to compare dynamic contrast-enhanced breast MRI at 7T and 3T. This is a relatively new subject, therefore we decided to present all information obtained in unilateral and bilateral coils. The use of a unilateral breast coil at 7T in the first 8 patients was subsequently replaced by a bilateral breast coil. This transition was a step forward towards clinical usage. However, further improvements are still necessary. By including multiple receiver elements in the bilateral coil, (van der Velden 2014) imaging with a high spatial and temporal resolution can be combined with the use of parallel imaging techniques. As mentioned previously, the feasibility of the implementation of a multiple receiver array has been shown by van den Bank et al. using a unilateral breast coil (van de Bank et al. 2013) and recently by Brown et al. in a bilateral set-up (Brown et al. 2013, 2014).

One limitation of our study is that T2w images were not acquired at 7T (and neither was in previous 7T breast MRI studies) (Brown et al. 2013; Gruber et al. 2014; Stehouwer et al. 2013a, b, 2014). Due to the use of multiple refocusing pulses, T2w sequences based in turbo spin echo have significant B1+ problems. Moreover, the association of the same multiple refocusing pulses and inversion recovery increases the SAR substantially (which is already close to the SAR limit at 3T). Fat-suppressed T2w sequences also have severe limitations (Gruber et al. 2014; Stehouwer et al. 2013a, b). Although the latest update of the BI-RADS lexicon recommends to include T2w imaging in the breast imaging protocol, (Edwards 2013) the most recent published T2w data at 7T do not meet imaging standards, (Umutlu et al. 2011) once we lack the necessary homogeneous distribution of 180º flip angles. Current developments such as RF pulses designed to compensate the linear decreasing B1+ field may enable accurate T2w imaging at 7T (van Kalleveen et al. 2015). However, further research and technical improvements are necessary (Gruber et al. 2014; van Kalleveen et al. 2015).

Another limitation is the small sample size. The first cohort study comparing breast MRI at 7T and 3T had a similar number of patients (24). Nevertheless, the presented lesions in our study encompassed a wide range of pathological diagnoses, including both benign and high risk lesions, and both in situ and invasive cancers.

## Conclusion

Our comparison study shows that 7T DCE breast MR allows BI-RADS conform analysis. However, technical improvements are needed (such as acquisition of T2w sequences and adjustment of B1+ field inhomogeneities) to further explore the clinical potential of 7T breast MRI.

## Methods

### Study population

The study population consisted of two groups. The first group consisted of female patients selected from a previously initialized 7T breast MRI feasibility study (Stehouwer et al. 2013b). These patients also underwent 3T MRI scan for clinical indications. The second group consisted of female patients who underwent both 3T and 7T MRI for the purpose of intra-individual comparison. In both groups the inclusion criteria were the same: women whose age was ≥18 years and with a suspicious breast lesion (BI-RADS 4 or 5) detected on conventional imaging. Exclusion criteria for both studies were: age <18 years, a history of surgery or radiotherapy on the ipsilateral breast, a Karnofsky score <70, pregnancy or lactation, and contra-indications to either MRI or administration of a gadolinium-based contrast agent.

Examinations at 7T and 3T were performed on separate days due to the administration of the contrast agent. In pre-menopausal patients, MRI was performed between days 6 and 13 of the menstrual cycle.

Both prospective studies were approved by the Institutional Review Board of the University Medical Center Utrecht, the Netherlands. Written informed consent was obtained from all patients before participation.

### Data acquisition

The 7T scans were performed on a whole-body scanner (Philips Healthcare, Cleveland, OH, USA). A unilateral two-channel (Stehouwer et al. 2013a, b; van de Bank et al. 2013) transmit/receive breast coil (MR Coils, Drunen, the Netherlands) was used for the first group. A bilateral four-channel (Italiaander and NPKOea 2012) coil (MR Coils, Drunen, the Netherlands) was used for the second group. The scan protocol included DCE (dynamic contrast-enhanced) imaging and an ultra-high resolution sequence. The DCE series was constructed using conventional imaging parameters comparable to 3T MRI. They consisted of seven consecutive 3D T1w gradient echo (GRE) sequences with fat suppression [TR/TE 5/2 ms, binomial nominal flip angle (FA) 15°, FOV 160 × 160 × 160/350 (unilateral/bilateral) mm^3^, acquired resolution 1 × 1 × 2 mm^3^, temporal resolution 63/67 s.], with the administration of 0.1 mmol/kg gadobutrol (Gd, Bayer Schering Pharma AG, Berlin, Germany). Ultra-high resolution imaging was performed using a T1w 3D GRE sequence with spectrally selective adiabatic inversion recovery (SPAIR) fat suppression [TR/TE/TI 7.0/2.9/120 ms, FA 12°, FOV 120 × 120 × 120/350 mm^3^, acquired resolution 0.5 mm isotropic].

For the first patient group, the 3T scans were performed on a whole-body scanner (Philips Healthcare, Best, the Netherlands), using a dedicated seven-channel receive-only breast coil (MRI devices, Würzburg, Germany). We used the hospital’s clinical tumor detection and staging protocol. This protocol included a T2-weighted (T2w) sequence, DCE imaging, and a high resolution sequence. The dynamic series consisted of six consecutive 3D T1w GRE sequences with SPAIR fat suppression [TR/TE/TI 3.1/1.17/90 ms, FA 10°, FOV 360 × 360 × 150 mm^3^, acquired resolution 1.1 × 1.1 × 2.4 mm^3^, temporal resolution 60 s], and with the administration of 0.1 mmol/kg gadobutrol. High resolution imaging was performed directly following the dynamic series [TR/TE/TI 4.5/1.67/90 ms, FA 10°, FOV 360 × 380 × 180, acquired resolution 0.65 × 0.65 × 2.00 mm^3^]. For the second patient group the protocol remained the same, except for the 3T dynamic series that was updated to obtain an acquired resolution of 0.9 × 0.92 × 1.80 mm^3^. Therefore, the previous high resolution sequence was discarded.

### Image analysis

Two observers (R1 and R2) independently rated all examinations. R1 was a breast radiologist with 8 years of experience in breast MRI, and R2 a radiologist with 3 years of experience. Both observers were blinded for the histopathological results.

We performed imaging analysis with Aegis breast software (Hologic Inc. MA), which enabled the observers to assess kinetic curve information and to use a color-coded overlay showing the different levels of initial and late enhancement. For the first group (with unilateral 7T images), the breast of interest was pointed out to the observers when assessing the 3T examination (for fair comparison).

First, image quality was rated for the dynamic series on a 5-point scale based on the following:

1. Excellent: no or hardly perceivable signal intensity variations across the field of view, i.e. homogeneous B1+ field, no or only mild artifacts, homogeneous fat suppression, and high visual SNR.
2. Good: mild heterogeneity changes in signal intensity across the field of view, e.g. mild gradual signal decrease from nipple towards chest wall, mild artifacts, mild inhomogeneity in fat suppression and high visual SNR.
3. Moderate: moderate B1+ inhomogeneity and/or moderate presence of artifacts and/or moderate inhomogeneous fat suppression and a high visual SNR.
4. Poor: insufficient signal intensity homogeneity, with substantial signal intensity variations across the field of view and/or substantial image degradation due to artifacts, substantial inhomogeneity of fat suppression and/or a low visual SNR.
5. Non-diagnostic: insufficient quality for diagnosis because of insufficient signal homogeneity or a complete loss of signal intensity in parts of the FOV owing to severe artifacts, severe inhomogeneity of fat suppression and/or poor visual SNR.

Second, all identified lesions were classified according to the first edition of the BI-RADS lexicon (2003) as proposed by the American College of Radiology (Molleran and Mahoney 2010). The maximal diameter of the identified mass lesions was measured.

Lastly, contrast-enhancement-to-noise ratios (CENRs) were calculated for mass lesions. Calculations were conducted by comparing the ratio of the signal intensity of the lesions and the standard deviation of the signal in a homogenous area of tissue adjacent to the lesion. This comparison was made between the pre and post-contrast administration images (Stehouwer et al. 2013a), resulting in a measurement that showed the lesion conspicuity.

### Statistics

Analyses were performed on a per examination basis. Image quality scores of both field strengths were compared using the Wilcoxon matched pairs signed rank test. Sensitivity, specificity, negative predictive value (NPV), and positive predictive value (PPV) with 95 % confidence intervals (CI) were calculated using dichotomized classification scores (lesions classified as BI-RADS 1–3 were considered benign, and the ones classified as 4–5 were considered malignant). The same scores were used to calculate inter-reader agreement of BI-RADS classification assessed with κ statistics. Histopathology was the reference standard. Sizes of malignant masses and CENRs were compared between field strengths using Wilcoxon matched pairs signed rank test. All analyses were performed using SPSS 20.0 (IBM Corp., NY, USA).
