# Erratum to: Dynamic contrast-enhanced breast MRI at 7T and 3T: an intra-individual comparison study

**DOI:** 10.1186/s40064-016-2514-9

**Published:** 2016-07-07

**Authors:** Gisela L. G. Menezes, Bertine L. Stehouwer, Dennis W. J. Klomp, Tijl A. van der Velden, Maurice A. A. J. van den Bosch, Floortje M. Knuttel, Vincent O. Boer, Wybe J. M. van der Kemp, Peter R. Luijten, Wouter B. Veldhuis

**Affiliations:** Department of Radiology and Nuclear Medicine, University Medical Centre Utrecht, P.O. Box 85500, 3508 GA Utrecht, The Netherlands

## Erratum to: SpringerPlus (2016) 5:13 DOI 10.1186/s40064-015-1654-7

Figures 1, 2, and 3 did not display on the HTML version of the original article. This has now been updated in the original article (Menezes et al. 2016) and the figures included below (Figs. 1, 2, 3).

**Fig. 1:**
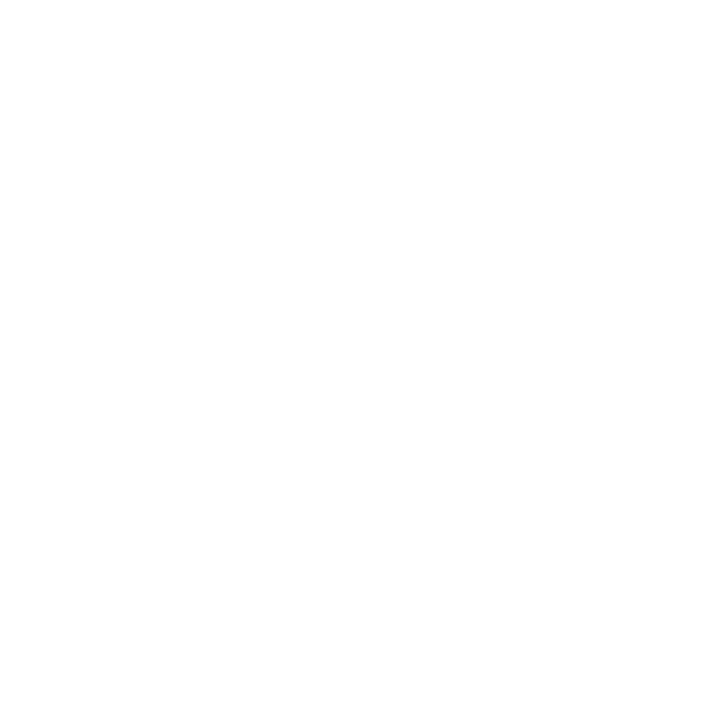
7T (**a**–**c**) and 3T (**d**, **e**) MRI images of a 67-year-old female with an invasive lobular carcinoma in her right breast. Transverse image of 2nd post contrast-injection series (**a**, **d**) shows an irregular mass lesion with spiculated margins (*arrows*) on both field strengths. *Inset* shows zoomed-in image. Ultra-high resolution 7T image of the same slice (**b**). The kinetic curve assessment showed an initial rapid rise and wash-out pattern in the delayed phase on both field strengths (**c**, **e**). Both observers rated the lesion as BI-RADS 5

**Fig. 2:**
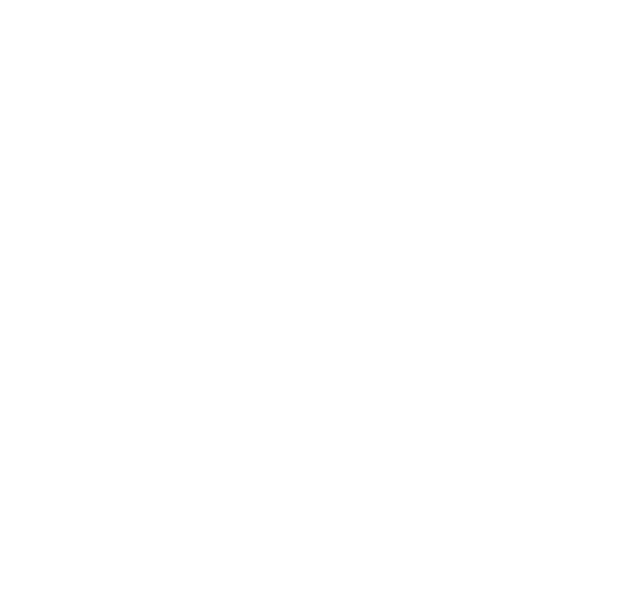
7T (**a**, **b**, **d**) and 3T (**c**, **e**) MRI images of a 65-year-old female patient. The depicted lesion in her right breast was diagnosed as fibrocystic changes after biopsy. Sagittal images of 2nd post contrast-injection series (**a**, **c**) show a lobular lesion (*arrow*) with irregular (R1 at 3T) or smooth margins (R1 at 7T and R2 at 3T and 7T). *Inset* shows zoomed-in image. Ultra-high resolution 7T image of the same slice (**b**). The kinetic curve assessment at 7T shows a rapid rise and persistent pattern in the delayed phase (**d**), and at 3T a rapid rise and plateau pattern (**e**). R1 classified the lesion as BI-RADS 3, and R2 as BI-RADS 3 (3T) and BI-RADS 2 (7T)

**Fig. 3:**
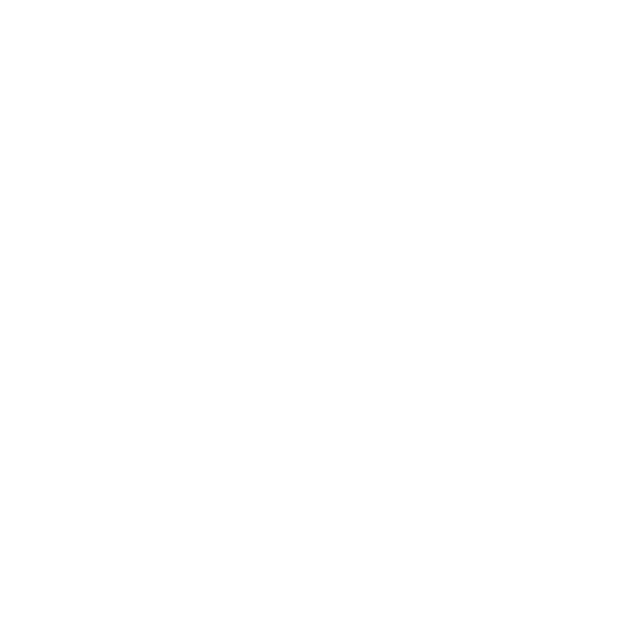
7T (**a**, **b**) and 3T (**c**, **d**) MRI results of a 47-year-old female patient with a history of inverted nipples. The biopsied index lesion in the right breast showed to be a cyst. Transverse image of 2nd post contrast-injection series (**a**, **c**). 7T MRI sagittal slice of high-resolution imaging (**b**), sagittal slice of 3T dynamic series at approximately the same location (**d**). At 7T MRI, diffuse non-mass-like enhancement was identified by R2, while R1 identified periductal enhancement (*arrow*). At 3T MRI, a focal non-mass-like enhancement was identified by R1 (*circle*), and multiple regions of non-mass-like enhancement were seen by R2. The observers rate the images BI-RADS 3 for 3T MRI, and BI-RADS 3 (R1) and 2 (R2) for 7T MRI
